# The influence of diabetes mellitus on the spectrum of uropathogens and the antimicrobial resistance in elderly adult patients with urinary tract infection

**DOI:** 10.1186/1471-2334-6-54

**Published:** 2006-03-17

**Authors:** Mario Bonadio, Silvia Costarelli, Giovanna Morelli, Tiziana Tartaglia

**Affiliations:** 1 Infectious Diseases Section, Department of Medicine, Ospedale S.Chiara, via Roma 56, 56100 Pisa, Italy

## Abstract

**Background:**

The role of Diabetes mellitus (DM) in the etiology and in the antimicrobial resistance of uropathogens in patients with urinary tract infection has not been well clarified. For this reason we have evaluated the spectrum of uropathogens and the profile of antibiotic resistance in both diabetic and non diabetic patients with asymptomatic urinary tract infection (UTI).

**Methods:**

Urinary isolates and their patterns of susceptibility to the antimicrobials were evaluated in 346 diabetics (229 females and 117 males) and 975 non diabetics (679 females and 296 males) who were screened for significant bacteriuria (≥10^5 ^CFU/mL urine). The mean age of diabetic and non diabetic patients was respectively 73.7 yrs ± 15 S.D. and 72.7 ± 24 (p = NS).

**Results:**

Most of our patients had asymptomatic UTI. The most frequent causative organisms of bacteriuria in females with and without DM were respectively : *E. coli *54.1% vs 58.2% (p = NS), *Enterococcus *spp 8.3% vs 6.5% (p = NS), *Pseudomonas *spp 3.9 vs 4.7% (p = NS). The most frequent organisms in diabetic and non diabetic males were respectively *E. coli *32.5% vs 31.4% (p = NS), *Enterococcus *spp 9.4% vs 14.5% (p = NS), *Pseudomonas *spp 8.5% vs 17.2% (p = <0.02). A similar isolation rate of *E. coli*, *Enterococcus *spp and *Pseudomonas *spp was also observed in patients with indwelling bladder catheter with and without DM. No significant differences in resistance rates to ampicillin, nitrofurantoin, cotrimoxazole and ciprofloxacin of *E. coli *and *Enteroccus *spp were observed between diabetic and non diabetic patients.

**Conclusion:**

In our series of patients with asymptomatic UTI (mostly hospital acquired), diabetes mellitus *per se *does not seem to influence the isolation rate of different uropathogens and their susceptibility patterns to antimicrobials.

## Background

Diabetes mellitus (DM) has long been considered to be a predisposing factor for urinary tract infection (UTI). However, since the concept of significant bacteriuria was introduced the reported data on the prevalence of asymptomatic bacteriuria appear to be conflicting [1-3].

Many UTIs are asymptomatic, especially in women. Unlike men with or without diabetes (among whom similar rates of UTI have been documented in most studies), several recent reports have noted a higher prevalence of asymptomatic bacteriuria among women with diabetes than among women without diabetes. However, other studies on asymptomatic outpatient diabetic women reported different results regarding the prevalence of bacteriuria [[4-17] table 1].

**Table 1:** Prevalence of bacteriuria in diabetic women reported in a series of selected studies

**Prevalence of bacteriuria**						
Reference N° year	Diabetics		Non diabetic		reported *P *value	Clinical features
	N° of pts	Pts with bacteriuria %	N° of pts	Pts with bacteriuria %		

**4 **1959	41	29.3	41	22	-	Hospitalised Asymptomatic
**5 **1966	128	18.8	114	7.9	-	Outpatients Asymptomatic
**6 **1967	195	2.0	n.a.		-	Schoolgirls Asymptomatic
**7 **1974	152	15.8	152	4.6	-	Outpatiens Asymptomatic
**8 **1984	92	15.2	91	11.0	NS	Outpatients, Asymptomatic/symptomatic
**9 **1986	341	9.1	100	5.0	<0.001	Outpatients Asymptomatic
**10 **1990	n.a.	31.3	n.a	16.0	< 0.05	Outpatients Asymptomatic
**11 **1992	147	17.7	n.a.		-	Outpatients, Asymptomatic
**12 **1993	239	6.3	236	3.4	NS	Outpatiens Asymptomatic
**13 **1995	1072	7.9	n.a.		-	Outpatiens Asymptomatic
**14 **2000	636	26.0	153	6.0	< 0.001	Outpatients Asymptomatic
**15 **2001	149	4.6	298	4.0	NS	Asymptomatic outpatients with gestational diabetes
**16 **2004	176	18.8	146	18.5	NS	Outpatients Asymptomatic
**17 **2005	363	9.6	350	2.9	-	Outpatients Asymptomatic

The most common cause of UTI in men and women with and without DM is *E. coli*. Some reports have noted that a lower proportion of UTIs is caused by this organism in diabetic patients as compared with age-matched non diabetic patients [12,18-21]. Antimicrobial resistance among uropathogens causing community and hospital acquired urinary tract infections is increasing [22]. Few data are available on the role of DM itself as a risk factor for the development of antimicrobial resistance of the uropathogens.

For over 10 years our unit has been involved in a program dedicated to the epidemiological surveillance of both symptomatic and asymptomatic urinary tract infections. Particularly, the local trends of the causative agents of urinary infections and their resistance patterns to the antimicrobials are being monitored. For this reason we have undertaken a study to evaluate the spectrum of the etiologic agents and their profiles of antimicrobial resistance on a large series of diabetic and non diabetic patients with UTI.

## Methods

Between March 1996 and June 2003, 10221 patients who were over the age of fifthy (6708 females and 3513 males) who were admitted to the department of medicine of the Pisa University-Hospital, were screened for asymptomatic bacteriuria (ASB). Out of the total studied population 1321 (12.9%) patients showed ASB. The criterion used for defining asymptomatic bacteriuria was the presence of at least 10^5 ^CFU/ml in 1 culture of clean-voided mid-stream urine specimen or obtained by urethral catheterisation.

Regarding the patients with ASB, urine samples were obtained by clean voided mid-stream technique in 1066 patients and by urethral catheter in 255 patients with an indwelling bladder catheter. All of the patients were admitted to the department of medicine of the hospital of Pisa as inpatients. One- hundred and fifty patients were transfered from surgical or urologic wards. One year prior to admittance 320 patients had a history of urethral catheterisation. Three-hundred-forty-six patients were diabetic (117 males and 229 females) and 975 non diabetic (296 males and 679 females).

Quantitative urine culture was performed using a dip-slide method; urine was also streaked on MacConkey agar. After an incubation at 37°C for 24 h, the microrganisms were identified by standard biochemical tests. In vitro susceptibility to antibiotics was performed by an agar diffusion method (Kirby Bauer) employing dried filter paper discs impregnated with specific concentration of antimicrobial agents in according to the National Committee for the Clinical Laboratory Standards. (Performance Standards for Antimicrobial Testing. Wayne, PA: NCCLS, 1995). All the patients with UTI were interviewed by the authors on the basis of a specific questionnaire in order to know their age, gender, presence or absence of symptoms of UTI.

The criteria used to exclude possible diabetic patients from non diabetic group was to make sure that they had a negative diabetic history and absence of glycosuria and fasting blood sugar less than 126 mg/dl.

The statistical analysis of the results was carried-out using X^2 ^test.

The research was conducted according to the Helsinki Declaration and was approved by the local ethic committee. Furthermore, all patients were informed about the aim of the study and their consent was obtained.

## Results

In the period between 1996 and 2003, 1321 (12.9%) out of 10221 patients (all older than 50 yrs;) who were admitted to the department of medicine of Pisa General hospital were found to have significant bacteriuria. Most of them had asymptomatic bacteriuria.

The rate of ASB was 12.76% (117 out of 917) and 11.4%(296 out of 2596) respectively in diabetic and non diabetic males. The rate of ASB was 14.97% (229 out of 1529) and 13.1% (679 out of 5175) in diabetic and non diabetic females respectively.

**Figure 1:**
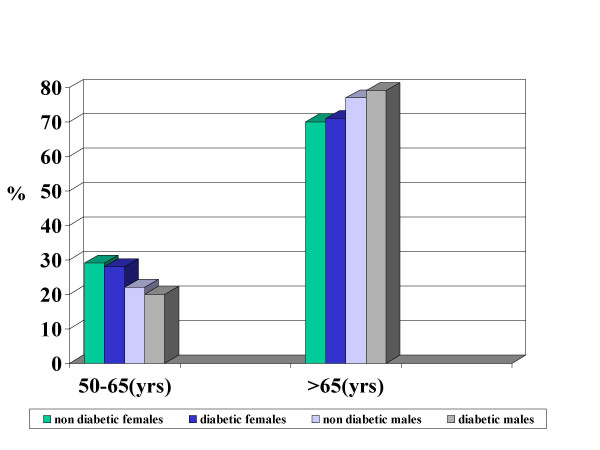
Distribution (%) of diabetic and non diabetic patients with urinary infection according to gender and age. Department of medicine, Pisa (1996–2003).

Regarding the patients with ASB, 346 patients (229 females and 117 males), had a diagnosis of diabetes mellitus (90% type II DM) and 975 (679 females and 296 males) were non diabetic patients. The mean HbA1c level of the diabetic patients at the time of admission was 7.8% ± 1.6 SD. The mean age of diabetic and non diabetic patients were respectively 73.7 yrs ± 15 SD and 72.7 ± 24 (p = .NS). The distribution of the diabetic and non diabetic patients with bacteriuria in according to the gender and age is represented in the figure 1. No statistical differences were found between diabetics and non diabetics in the different groups of age and gender; Seventy-eight (22.5%) out of 346 diabetics and 177 (18.1%) out of 975 non diabetics had an indwelling bladder catheter respectively.

*E. coli *was the most frequent uropathogen isolated, and was responsible for UTI in 32.5% of diabetic and 31.4% of non diabetic males (p = NS). *Enterococcus *spp was isolated in 9,4% vs 14,5% (p = NS), *Pseudomonas *spp was isolated in 8.5% vs 17.2% (p = 0.02) of diabetic and non diabetic patients respectively (Tab. 2). *E. coli *was more frequent in women (diabetics 54.1% and non diabetics 58.2%) than in men (diabetics 32.5% and non diabetics 31.4%). The isolation rate of *Enterococcus *spp (8.3% vs 6.5%. p = NS) and *Pseudomonas *spp (3.9% vs 4.7%, p = NS) was also similar in diabetic and non diabetic women (Tab. 3).

**Table 2:** Isolation rate of uropathogens in male patients with and without diabetes. Department of Medicine, Pisa (1996–2003)

	Diabetic males		Non diabetic males		
	N°	%	N°	%	P value

*E. coli*	38	32.5	93	31.4	NS
*Enterococcus *spp	11	9.4	42	14.5	NS
*Pseudomonas *spp	10	8.5	51	17.2	0.02
Other	58	49.5	110	37.2	NS
Total	117	100	296	100	

**Table 3:** Isolation rate of uropathogens in female patients with and without diabetes. Department of Medicine, Pisa (1996–2003)

	Diabetic females		Non diabetic females		
	N°	%	N°	%	p value

*E. coli*	124	54.1	395	58.2	NS
*Enterococcus *spp	19	8.3	44	6.5	NS
*Pseudomonas *spp	9	3.9	32	4.7	NS
Other	77	33.6	208	30.6	NS
Total	229	100	679	100	

The separate evaluation of women with symptomatic bacteriuria demonstrated, in both diabetic and non diabetic females with UTI, a similar frequency of the different strains (*E. coli *: 54% vs 58.2%, p = NS, *Enterococcus *spp 8.3% vs 6.5%, p = NS, *Pseudomonas *spp 3.9% vs 4.7%, p = NS).

The rates of uropathogens in diabetic and non diabetics patients (males plus females) with indwelling bladder catheter were respectively: *E. coli *30.8% vs 24.9%, p = NS ; *Enterococcus *spp 21.8% vs 16.45 %, p = NS; *Pseudomonas *spp 12.8% vs 18.1%, p = NS (Tab. 4).

**Table 4:** Isolation rate of uropathogens in diabetic and non diabetic patients with indwelling bladder catheter. Department of Medicine, Pisa (1996–2003)

	Diabetic patients with indwelling catheter		Non diabetic patients with indwelling catheter		
	N°	%	N°	%	p value

*E. coli*	24	30.8	44	24.9	NS
*Enterococcus *spp	17	21.8	29	16.4	NS
*Pseudomonas *spp	10	12.8	32	18.1	NS
Other	27	34.6	72	40.0	NS
Total	78	100	177	100	

The rates of antibiotic resistance of *E. coli *in diabetic vs non diabetic patients were : ampicillin 29% vs 30.6%, p = NS; cotrimoxazole 19.2% vs 17.4%, p = NS; ciprofloxacin 11.6% vs 6.6%, p = NS ; nitrofurantoin 8.4% vs 6.9%, p = NS (Tab. 5). The *Pseudomonas *strains isolated in diabetic and non diabetic patients had similar patterns of resistance against antipseudomonas drugs: ciprofloxacin 50% vs 55.4%, p = NS; ceftazidime 41.1% vs 14.1%, p = NS: imipenem 16.6% vs 11.1%, p = NS; amikacin 16.6% vs 14.6%, p = NS.

**Table 5:** Antimicrobial resistance of urinary *E. coli *in patients with and without diabetes. Department of Medicine, Pisa (1996–2003).

	Diabetic patients			Non diabetic patients			
Antomicrobial agent	Tested strains	Resistant strains		Tested strains	Resistant strains		

	n°	n°	%	n°	n°	%	p values

ampicillin	157	46	29	490	147	39.6	NS
cotrimoxazole	151	29	19.2	441	77	17.4	NS
ciprofloxacin	154	18	11.6	463	31	6.6	NS
nitrofurantoin	178	15	8.4	495	34	6.9	NS

## Discussion

In this study we have tried to determine whether there are differences in the bacteriologic patterns of UTI and in the antibiotic sensitivity patterns of the pathogens concerned with diabetic and non-diabetic patients. The study was carried-out on a large series of elderly adult diabetic and non diabetic patients admitted to the medical wards. More than 70% of the patients were older than 65 yrs of age. The age and the gender were absolutely comparable in both study populations as well as the proportion of patients with indwelling bladder catheter. The rate of E. coli isolation we found in both diabetic and non diabetic patients was much lower than that usually observed in community acquired UTI, thus suggesting that a significant part of our patients had nosocomial acquired UTI. Other studies have found that urinary Klebsiella is more frequent in patients with DM than in non diabetic patients [22-24].

Some authors have defined UTI in patients with DM as complicated when the UTI is symptomatic [23,24]. The spectrum of uropathogens we found in our patients and patterns of antimicrobial resistance in both DM and non DM patients are similar to those observed in other studies dealing with complicated UTI's. This finding can be explained by the fact that 20% of our patients had an indwelling bladder catheter. In addition many of our patients may have undergone previous antimicrobial treatment.

When our patients with indwelling catheter were considered separately, the rate of the different uropathogens did not differ significantly in diabetic and non diabetic groups. The spectrum of uropathogens and antimicrobial pattern resistance we found in our series of patients with catheter associated UTI may be different from those observed in other hospitals. This may depend on the different policy of antibiotics used in the various hospitals.

It is interesting to note that in a clinical setting different from ours, urinary isolates of symptomatic ambulatory postmenopausal women did not show a significant difference in the bacterial species when compared to a matched group of women without DM [25]. We observed a higher isolation rate of *Pseudomonas *spp in non diabetic than that in diabetic males; therefore we must underline that many of our male non diabetic patients had a history of a previous instrumentation of the urinary tract.

Regarding the antimicrobial resistance profile of the uropathogens, we observed that the isolated *E. coli *strains were resistant at similar rates to ampicillin, cotrimoxazole, ciprofloxacin and nitrofurantoin in both diabetic and non diabetic patients. The high rate of *E. coli *resistance to ampicillin and cotrimoxazole we found in our series precludes, at least in our area, the choice of these or similar drugs in the empirical initial treatment of adult hospitalized patients with UTI. In a study performed in an emergency department, an association was found between the presence of cotrimoxazole resistance and diabetes, recent hospitalization and recent use of the same drug [26] but in an out-patients setting no correlation was found between *E. coli *resistance to cotrimoxazole [27,28] or to quinolones and diabetes mellitus.

The proportion of *Pseudomonas *resistance to ciprofloxacin was very high as previously reported in our hospital but at a similar rate in patients with and without DM.

## Conclusion

We found a low proportion of *E. coli *isolates (especially in men) in hospitalized elderly adult patients with asymptomatic UTI in both diabetics and non diabetics. In addition, the resistance of the uropathogens to the antibiotics was similar in patients with and without DM. These results confirm our previous observations obtained on a smaller size sample of patients with and without DM [29].

In our series of patients with asymptomatic UTI (mostly hospital acquired), diabetes mellitus could not be considered *per se *a risk factor for the emergence of a non *E. coli *organism and for antibiotic resistance.

## Competing interests

The author(s) declare that they have no competing interests.

## Authors' contributions

MB conceived of the study, coordinated the activity of the microbiology laboratory and the collection of clinical data and wrote the final draft.

SC cooperated in the collection of the clinical data, in the validation of the microbiologic results and performed the statistical evaluation.

GM cooperated in the collection of the clinical data and in the validation of the microbiologic results.

TT cooperated in the collection of the clinical data and in the validation of the microbiologic results.

All the authors read and approved the final manuscript.

## Pre-publication history

The pre-publication history for this paper can be accessed here:


